# Oral Administration of *Lactobacillus gasseri* and *Lacticaseibacillus rhamnosus* Ameliorates Amyloid Beta (Aβ)-Induced Cognitive Impairment by Improving Synaptic Function Through Regulation of TLR4/Akt Pathway

**DOI:** 10.3390/antiox14020139

**Published:** 2025-01-24

**Authors:** Hye Ji Choi, Hyo Lim Lee, In Young Kim, Yeong Hyeon Ju, Yu Mi Heo, Hwa Rang Na, Ji Yeon Lee, Soo-Im Choi, Ho Jin Heo

**Affiliations:** 1 Division of Applied Life Science (BK21), Institute of Agriculture and Life Science, Gyeongsang National University, Jinju 52828, Republic of Korea; hjchoi0820@gnu.ac.kr (H.J.C.); gyfla059@gnu.ac.kr (H.L.L.); inzero331@gnu.ac.kr (I.Y.K.); ju8172001@gnu.ac.kr (Y.H.J.); yumi@gnu.ac.kr (Y.M.H.); hrna@gnu.ac.kr (H.R.N.); 2 MEDIOGEN, Co., Ltd., Bioballey 1-ro, Jecheon-si 27159, Republic of Korea; ljy@mediogen.co.kr (J.Y.L.); csi@mediogen.co.kr (S.-I.C.)

**Keywords:** *Lactobacillus gasseri*, *Lacticaseibacillus rhamnosus*, cognitive function, neuroinflammation, synaptic function

## Abstract

This study investigated the anti-amnesic effects of *Lactobacillus gasseri* (*L. gasseri*) MG4247 and *Lacticaseibacillus rhamnosus* (*L. rhamnosus*) MG4644 in amyloid beta (Aβ)-induced mice. We confirmed that oral administration of *L. gasseri* MG4247 and *L. rhamnosus* MG4644 ameliorated cognitive impairment in Aβ-induced mice using Y-maze, passive avoidance, and Morris water maze tests. Oral administration of *L. gasseri* MG4247 and *L. rhamnosus* MG4644 protected the antioxidant system by regulating superoxide dismutase levels, reduced glutathione levels, and reduced malondialdehyde contents. Similarly, they attenuated mitochondrial function by decreasing mitochondrial reactive oxygen species levels and increasing mitochondrial membrane potential and ATP levels. In addition, they regulated neuroinflammation and neurotoxicity by modulating the Toll-like receptor 4 (TLR4)/protein kinase B (Akt) pathway. As a result, they enhanced synaptic function by regulating acetylcholine contents, acetylcholinesterase activity, and the expression of synaptic-function-related proteins such as AChE, ChAT, SYP, PSD-95, and GAP-43. Furthermore, the administration of *L. gasseri* MG4247 and *L. rhamnosus* MG4644 improved dysbiosis by promoting the growth of beneficial bacteria while suppressing the growth of harmful bacteria. Therefore, these results suggest that *L. gasseri* MG4247 and *L. rhamnosus* MG4644 may be used as probiotics to prevent cognitive impairment.

## 1. Introduction

Alzheimer’s disease (AD), including a serious cognitive dysfunction, is a degenerative neurological disease in which cognitive functions such as judgment, memory, and reasoning gradually deteriorate as brain neurons gradually decline [1]. The annual expenditure on treating AD amounts to USD 1 trillion and is expected to increase twofold by 2030 [2]. AD is a multifactorial disease caused by various factors and has a variety of causes, such as the accumulation of toxic amyloid beta (Aβ), hyperphosphorylated tau protein, oxidative stress, mitochondrial dysfunction, and neuroinflammation [3]. Among the various causes, Aβ is derived from amyloid precursor protein through the enzymatic activity of β- and γ-secretase, which subsequently forms various soluble species that transition into crossed β-sheet fibers to develop plaques [4]. In particular, the formed Aβ plaques are highly toxic and are reported to affect synapses, which are fundamental to the processes of learning and memory [5]. The shape and function of these synapses can change depending on the efficiency of neurotransmission, which is called synaptic plasticity [6]. As AD progresses, this synaptic plasticity results in dendrite shrinkage and collapse due to excessive Aβ production, and the reduction in synaptic plasticity impairs learning and memory abilities [5]. Aβ plaques interact with Toll-like receptor 4 (TLR4), classified as a pattern recognition receptor located at microglial cell surfaces, initiating downstream inflammatory signaling pathways leading to neuroinflammation in AD [7]. This inflammatory response suppresses the activation of protein kinase B (Akt), a crucial factor for maintaining neuron survival and synaptic functionality within intracellular signaling pathways [8]. Simultaneously, glycogen synthase kinase-3 beta (GSK-3β), a key enzyme linked to tau hyperphosphorylation and neuron death, becomes excessively active [9]. Due to this inflammatory response, the decrease in Akt expression and the increase in GSK-3β expression cause tau hyperphosphorylation and neuronal apoptosis, which induce a vicious cycle that promotes Aβ production and accumulation [10]. Excessive free radicals generated by Aβ plaques induce oxidative stress, which drives the production of reactive oxygen species (ROS) and causes impairment of mitochondria, including ATP depletion and neuron apoptosis due to electron transport chain disruption [11]. As a result, neuroinflammation, neurotoxicity, and decreased synaptic plasticity occur, which are major features of neurodegenerative diseases such as AD. Therefore, TLR4 activation and Akt pathway abnormalities caused by Aβ accumulation result in neuroapoptosis and reduced synaptic plasticity from inflammation in the nervous system and oxidative damage, key pathological characteristics of AD [7]. Modulation of TLR4 and Akt pathways may be important therapeutic targets for alleviating neuroinflammation and cell survival.

Recent research examined associations between gut microbiota (GMB) and cognitive function [12,13,14]. The gut microbiota comprises countless protozoa, bacteria, and viruses, serving a vital role in controlling systemic inflammation and strongly affecting disease progression [12,13,14]. Alterations in gut microbiota can disrupt balance, triggering cognitive dysfunction by promoting neuroinflammation and enhancing inflammatory factor release due to elevated levels of harmful bacteria [5]. In particular, GMB imbalance promotes the growth of specific bacterial strains that produce amyloid fibrils, and bacterial amyloids from these strains can penetrate the blood–brain barrier (BBB) to activate TLR4 in the central nervous system (CNS) of the brain [15]. This amplifies the neuroinflammatory response and promotes Aβ fibril formation, a key mechanism of AD pathology [7,12]. Therefore, modulating the GMB may be a good therapeutic strategy to improve cognitive dysfunction induced by Aβ.

Probiotics, described as “live microorganisms that, when administered in adequate amounts, provide health benefits to the host”, have traditionally included *Lactobacillus* and *Bifidobacterium* species as representative strains [16]. Recently, it has been reported that the ingestion of probiotic strains such as *Lactobacillus* and *Bifidobacterium* can induce changes in GMB that can change the metabolites produced by GMB and affect brain function and behavior through the gut–brain axis [13]. In particular, lactic acid bacteria (LAB) intake reconstitutes GMB, restores physiological and psychological deficits, and reduces cognitive and behavioral changes caused by stress [12]. Moreover, *L. gasseri* is known to reside in the gastrointestinal tract and prevent pathogenic bacteria from adhering to cells [17], while *L. rhamnosus* is known to adhere to intestinal epithelial tissue and persist, thereby strengthening cytoskeletal integrity [16]. According to Yun et al., *L. gasseri* NK109 reduced neuroinflammation by enhancing brain-derived neurotrophic factor (BDNF) levels in the hippocampal region in mice with cognitive impairment induced by *Escherichia coli* K1 [18]. Moreover, *L. gasseri* MG4247 exerted anti-inflammatory effects by regulating the levels of type 1 T helper (Th1) cells and type 2 T helper (Th2) cells in mast cells and macrophages [19]. *L. rhamnosus* JB-1 has been reported to elevate γ-aminobutyric acid (GABA) mRNA, an essential inhibitory neurotransmitter within the CNS, in the cerebral cortex region of mice with stress-related-disorders-induced depression [20]. Therefore, we evaluated the effects of cell-free supernatants (CFSs) from *L. gasseri* MG4247, *L. rhamnosus* MG4644, *L. rhamnosus* MG4643, and *L. rhamnosus* MG4289 on cell viability and ROS production levels in H_2_O_2_-induced HT22 and SK-N-MC cells (Figure S1). A CFS used in the experiment refers to the supernatant obtained by centrifuging the culture solution after culturing LAB. The experimental results showed that all CFSs of four LAB treatments exhibited effective cytoprotective effects. According to this, we chose *L. gasseri* MG4247 and *L. rhamnosus* MG4644 for animal study, considering the industrialization potential as functional food materials. In previous studies, the CFS of LAB was used to verify their effectiveness, but in this study, live LAB were used to confirm more practical applicability. Therefore, in this study, we evaluated the cognitive function improvement effect of *L. gasseri* MG4247 and *L. rhamnosus* MG4644 in Aβ-induced mice and confirmed the possibility of industrializing them as probiotic materials.

## 2. Materials and Methods

### 2.1. Preparation of Probiotics

The *L. gasseri* MG4247 and *L. rhamnosus* MG4644 which were used in this study were provided by Mediogen Co., Ltd. (Jecheon, Republic of Korea). The strains originated from humans, and the identified information is in Table S1. After incubating in an anaerobic chamber at 37 °C for 18 h, the strain pellets were centrifuged. The pellets of strains were harvested and freeze-dried. Mediogen Co., Ltd. supplied the powdered probiotic samples used in the experiment.

### 2.2. Animal Design

The Aβ-induced AD model can reproduce the pathological features of AD, such as synaptic dysfunction and neuroinflammation, in a short period of time [21]. In addition, it has been reported as a successful method for establishing an AD animal model [22], so the Aβ-induced AD model was used in this study. Male ICR mice (4 weeks old) were purchased from Koatech (Pyeongtaek, Republic of Korea), acclimated for 1 week in a basic rearing environment (temperature: 22 ± 2 °C, humidity: 55%, and light/dark cycle: 12 h), and provided unlimited food and water intake freely. All mice were maintained under identical environmental conditions, and consistent procedures were followed for handling between experiments to minimize stressors. They were then randomly divided into four groups (normal control (NC), Aβ, MG4247, and MG4644), and each group consisted of 13 mice. The NC group and the Aβ group were administered phosphate-buffered saline (PBS), and the MG4247 and MG4644 groups were administered orally daily with 1 × 10^9^ CFU/200 μL of *L. gasseri* MG4247 and *L. rhamnosus* MG4644, respectively, for three weeks. Three weeks later, the NC group was injected with 0.89% NaCl, and the Aβ, MG4247, and MG4644 groups were injected with 410 pM Aβ_1-42_ (PP69, Sigma-Aldrich Chemical Co., St. Louis, MO, USA) in 0.89% NaCl at 2.5 mm bregma using a micro-Hamilton syringe with a 26-gauge needle. All oral administration and injection procedures were performed under identical conditions. After a three-day recovery period, mouse behavioral experiments were performed, after which the mice were sacrificed using CO_2_. This study was conducted following approval by the Ethics Committee for Animal Experiments at Gyeongsang National University (approval number: GNU-240129-M0030; approved on 29 January 2024).

### 2.3. Behavioral Test

#### 2.3.1. Y-Maze Test

The Y-maze test was performed to measure short-term and working memory. Mice were placed on one arm of the Y-maze (33 × 15 × 10 cm) and monitored for 8 min using the program (SMART 3.0, Panlab, Barcelona, Spain). The number of times the mouse entered the 3 arms consecutively without repeating them was defined as the ability to cross behavior.

#### 2.3.2. Passive Avoidance Test

The passive avoidance test was performed to evaluate short-term and long-term memory for fear stimuli. The experiment was conducted in a chamber divided into light and dark areas, and on the first day, when the mouse’s four paws entered the dark area, the passage was blocked, and an electric stimulus of 0.5 mA was applied for 3 s. At 24 h later, the same process as on the first day was performed, and the time it took for the mouse to enter the dark chamber with all four paws was measured up to 300 s.

#### 2.3.3. Morris Water Maze (MWM) Test

The MWM test assessed spatial cognition and long-term memory. This experiment was conducted in a circular water pool (150 cm diameter × 60 cm height) for six days. The MWM test was recorded using the program (SMART 3.0, Panlab). On the first day, the escape platform was visible about 1 cm away, and the mice were given 1 min to learn to find each quadrant (W, E, S, N). If the mice reached the escape platform (located in the W zone) within 1 min, the mice were allowed to adapt for 10 s. If the mice could not find the escape platform, they could reach it by pointing to it with hand, allowing them to adapt for 20 s. From the second to the fourth day, the escape platform was hidden underwater and the time it took the mice to find the platform within 1 min was recorded. Finally, on the sixth day, the escape platform was removed from the MWM pools, and the duration mice remained in the zone previously occupied by the platform was recorded.

### 2.4. Antioxidant System

To measure the SOD levels in brain tissue, the brain tissue was mixed with PBS, homogenized, and processed at 4 °C by centrifuging at 400× *g* for 10 min. The SOD contained in the pellet was obtained by mixing it with an SOD extraction buffer consisting of distilled water, triton X-100, 0.2 M phenylmethylsulfonyl fluoride (PMSF), and water-soluble tetrazolium (WST) solution, kept on ice for 30 min, and then processed at 4 °C by centrifuging at 10,000× *g* for 10 min. SOD contained in the supernatant was measured using a SOD kit (Dojindo, Kumamoto, Japan) according to the manufacturer’s instructions.

To assess reduced GSH levels in brain tissue, the brain tissue was homogenized with 1 mM ethylenediamine tetraacetic acid (EDTA) and 10 mM sodium phosphate buffer (pH 6–7) at 4 °C by centrifuging at 10,000× *g* for 15 min. The equal ratio was mixed with supernatant and 5% metaphosphoric acid at 4 °C by centrifuging at 2000× *g* for 2 min. The supernatant was reacted with o-phthaldialdehyde (OPT), 0.26 M Tris-HCl (pH 7.6), and 0.65 N sodium hydroxide at room temperature for 15 min. Then, the reactants were measured at an excitation wavelength of 360 nm and an emission wavelength of 430 nm using a fluorescence microplate reader (Infinite F200, Tecan, Mannedorf, Switzerland).

To determine MDA contents in brain tissue, the brain tissue with PBS added was homogenized and processed at 4 °C by centrifuging at 2456× *g* for 10 min. The supernatant was mixed with 1% phosphoric acid and 0.67% thiobarbituric acid (TBA), incubated with shaking at 95 °C for 1 h, and cooled and processed at 4 °C by centrifuging at 5000× *g* for 1 min. After that, the supernatant was measured at 532 nm using a spectrophotometer (UV-1800, Shimadzu, Kyoto, Japan).

### 2.5. Mitochondrial Function

To evaluate mitochondrial function, mitochondria were extracted from brain tissue as in the study of Go et al. [23].

To measure the ROS levels in mitochondrial extracts, respiration buffer (0.13 M KCl, 2 mM KH_2_PO_4_, 2.5 mM malic acid, 0.02 M HEPES, 1 mM MgCl_2_, 5 mM pyruvate, and 0.5 M EGTA) (pH 7.0) with 2′,7′-dichlorofluorescin diacetate (DCF-DA) was reacted with the mitochondrial extract. Then, the reactants were measured at an excitation wavelength of 360 nm and an emission wavelength of 430 nm using a fluorescence microplate reader (Infinite F200, Tecan).

To assess the mitochondrial membrane potential (MMP) of mitochondrial extracts, the mitochondrial extracts were reacted with mitochondrial isolation buffer containing 5 mM malic acid, 5 mM pyruvate, and 0.001 mM 1,1′,3,3′-tetraethyl-5,5′,6,6′-tetrachloroimidacarbocyanine iodide (JC-1). The reactants were then measured at an excitation wavelength of 535 nm and an emission wavelength of 590 nm using a fluorescence microplate reader (Infinite F200, Tecan).

To determine the ATP contents of the mitochondrial extract, it was centrifuged at 4 °C at 13,000× *g* for 10 min. The pellet was then treated with 0.02 M Tris-acetate buffer containing 1% trichloroacetic acid (pH 7.75) and centrifuged at 4 °C at 10,000× *g* for 15 min. Mitochondrial ATP-containing supernates were measured using an ATP Kit (Promega, Madison, WI, USA).

### 2.6. Cholinergic System

To determine acetylcholine (ACh) contents in brain tissue, the brain tissue with PBS added was homogenized and processed at 4 °C by centrifuging at 13,572× *g* for 30 min. The supernatant was mixed with 2 M hydroxylamine-HCl and 3.5 N sodium hydroxide for 1 min. After that, 0.5 N HCl was added to the reaction solution, and then 0.37 M FeCl_3_·6H_2_O containing 0.1 N HCl was added and reacted. The reaction solution was measured at 37 °C at 540 nm using a microplate reader (Epoch2, BioTek Instrument Inc., Winooski, VT, USA).

To measure acetylcholinesterase (AChE) activity in brain tissue, the brain tissue with PBS added was homogenized and processed at 4 °C by centrifuging at 13,572 ×g for 30 min. The supernatant was mixed with 0.05 M sodium phosphate buffer and incubated at 37 °C for 15 min. Afterwards, 1 mM 5,5′-dithiobis-2-nitrobenzoic acid (DTNB) and 0.5 mM acetylthiocholine were added and it was incubated at 37 °C for 15 min. The reaction solution was measured at 37 °C at 405 nm using a microplate reader (Epoch2, BioTek).

### 2.7. Western Blot

The Western blot was performed according to the study of Lee et al. and used whole brain tissue [24]. The primary and secondary antibodies used in the experiment are shown in Table S2.

### 2.8. 16S rRNA Sequencing

Fresh fecal samples were collected using the AccuStool Collection Kit (AccuGene, Incheon, Republic of Korea) and kept at −80 °C until further analysis. Fecal DNA extraction was performed with the AccuFAST automation system (AccuGene Inc., Incheon, Republic of Korea) following the manufacturer’s protocol. For MiSeq sequencing, bacterial genomic DNA was amplified using primers targeting the V3-V4 hypervariable regions of the 16S rRNA genes. The primers were used in sequencing 341F (5′-CCTACGGGNGGCWGCAG-3′) and 805R (5′-GACTACHVGGGTATCTAATCC-3′). Amplification of the 16S rRNA genes involved 25 cycles of polymerase chain reaction (PCR) using the KAPA HiFi HotStart ReadyMix (Roche sequencing, Basel, Switzerland). The PCR products (~600 bp) were purified using HiAccuBeads for NGS library preparation (AccuGene Inc.). Equimolar pooling of amplicon libraries was conducted, followed by sequencing on an Illumina MiSeq system with the MiSeq Reagent Kit v3 (600 cycles, Illumina, San Diego, CA, USA). Quality assessment of raw fastq files was performed with FastQC (version 0.11.9). Microbiome data processing and analysis were performed using the QIIME2 platform, with quality control and denoising completed through DADA2. Taxonomic assignment of ASVs was conducted using the SILVA database (version 138.1) to characterize taxonomic distributions.

### 2.9. Statistical Analysis

Experimental results were performed using SAS software (Ver. 9.4, SAS Institute, Cary, NC, USA). Data were expressed as mean ± standard deviation unless otherwise stated. Statistical analysis was performed using Duncan’s multiple range test and one-way ANOVA, and *p* < 0.05 was considered statistically significant. Additionally, Pearson’s correlation between gut microbiota and key factors related to cognitive function was performed using RStudio (Ver. 4.4.2) and visualized as a heat map.

## 3. Results

### 3.1. L. gasseri MG4247 and L. rhamnosus MG4644 Ameliorate Memory and Learning Ability

**Figure 1:**
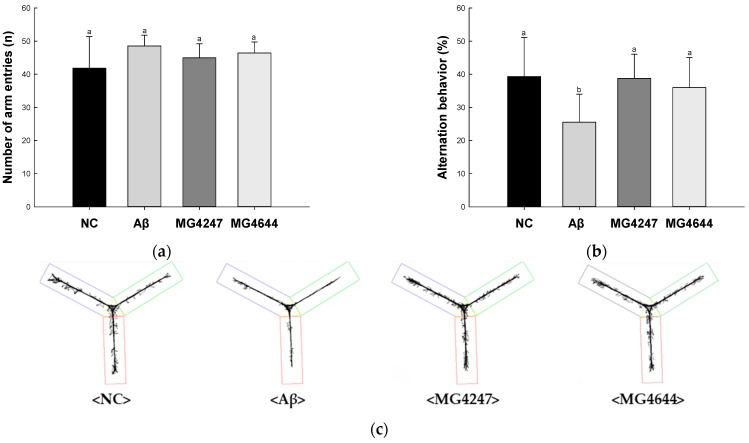

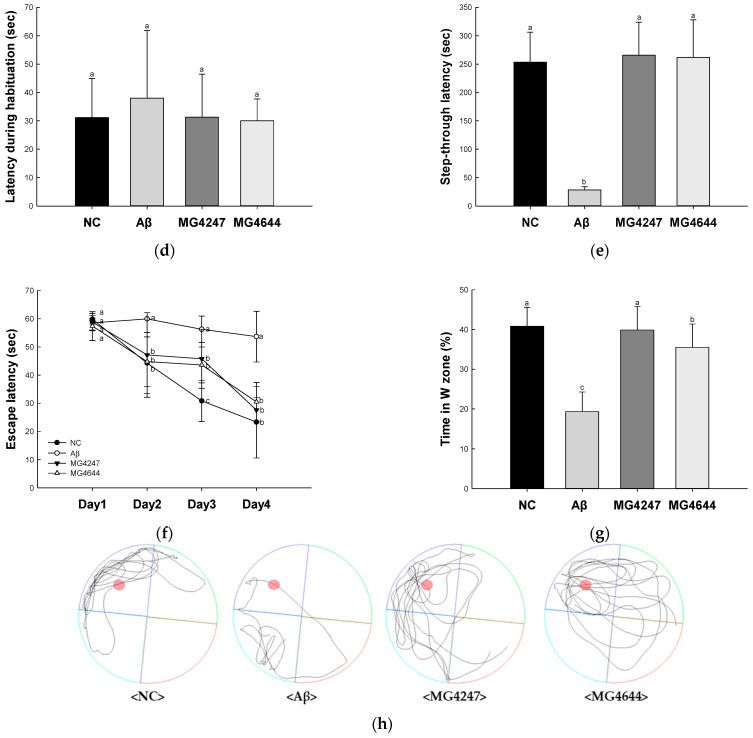
Effects of *L. gasseri* MG4247 and *L. rhamnosus* MG4644 on memory and learning ability in Aβ-induced cognitive dysfunction mice. The number of arm entries (**a**), alternation behavior (**b**), and 3D images (**c**) on the Y-maze test, latency during habituation (**d**) and step-through latency (**e**) on the passive avoidance test, and escape latency (**f**) during the hidden test and time in W zone (**g**) during the probe test and swimming pattern visualization image (**h**) on the Morris water maze test. Data are presented as mean ± standard deviation (*n* = 7). Lowercase letters in the histograms indicate statistical differences between groups (*p* < 0.05). The paths the mice moved were marked in black, and the other colors were used to distinguish areas (**c**,**h**).

In the Y-maze test (Figure 1a–c), the number of arm entries among all groups showed no difference (Figure 1a). Alternation behavior was decreased in the Aβ group (25.52%) compared to the NC group (39.32%). However, the alternation behavior of the MG4247 and MG4644 groups (38.73% and 36.01%) was increased as compared to the Aβ group.

In the passive avoidance test (Figure 1d,e), the latency during habituation among all groups showed no difference on the first day (Figure 1d). Step-through latency was decreased in the Aβ group (28.43 s) compared to the NC group (253.43 s) on the second day. However, the step-through latency of the MG4247 and MG4644 groups (265.71 s and 261.86 s) was increased in comparison to the Aβ group on the second day.

In the MWM test (Figure 1f–h), the escape latency among all groups showed no difference on the first day (Figure 1f). Afterwards, as the experiment continued for three days, the escape time in the NC group decreased, but there was no significant difference in the escape time in the Aβ group. The escape time of the MG4247 and MG4644 groups decreased compared to the Aβ group over the experimental period. On the last day of the experiment, the platform was removed and the time the mice spent in the location of the platform (W zone) was measured. The Aβ group (19.33%) decreased the time spent in the W zone compared to the NC group (40.82%). The MG4247 and MG4644 groups (39.87% and 35.51) increased the time spent in the W zone compared to the Aβ group.

### 3.2. L. gasseri MG4247 and L. rhamnosus MG4644 Enhance Antioxidant Activity

**Figure 2:**
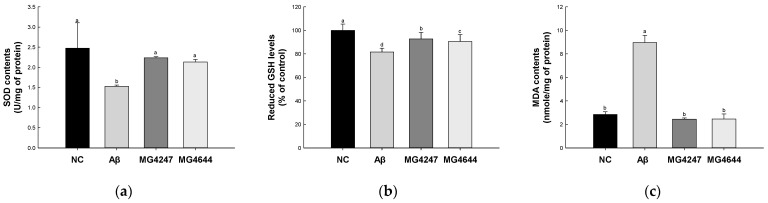
Effects of *L. gasseri* MG4247 and *L. rhamnosus* MG4644 on antioxidant system of brain tissue in Aβ-induced cognitive dysfunction mice. Superoxide dismutase (SOD) contents (**a**), reduced glutathione (GSH) levels (**b**), and malondialdehyde (MDA) contents (**c**). Data are presented as mean ± standard deviation (*n* = 5). Lowercase letters in the histograms indicate statistical differences between groups (*p* < 0.05).

SOD contents and reduced GSH levels of the Aβ group (1.53 U/mg of protein and 81.64%, respectively) were decreased compared to the NC group (2.47 U/mg of protein and 100%, respectively) (Figure 2a,b). However, the SOD contents and reduced GSH levels of the MG4247 (2.23 U/mg of protein and 92.79%, respectively) and MG4644 (2.13 U/mg of protein and 90.58%, respectively) groups were increased compared to the Aβ group.

MDA contents of the Aβ group (8.97 nmole/mg of protein) were increased compared to the NC group (2.85 nmole/mg of protein) (Figure 2c). However, the MDA contents of MG4247 and MG4644 (2.45 and 2.45 nmole/mg of protein, respectively) groups were decreased compared to the Aβ group.

### 3.3. L. gasseri MG4247 and L. rhamnosus MG4644 Improve Mitochondrial Function

**Figure 3:**
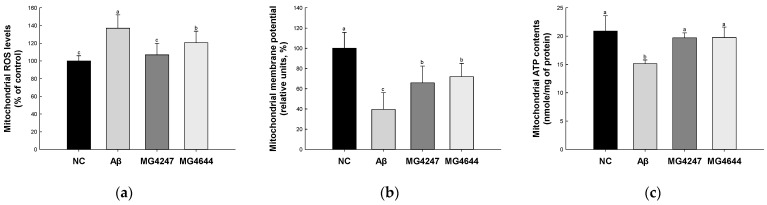
Effects of *L. gasseri* MG4247 and *L. rhamnosus* MG4644 on mitochondrial function of brain tissue in Aβ-induced cognitive dysfunction mice. Mitochondrial ROS levels (**a**), mitochondrial membrane potential (**b**), and mitochondrial ATP contents (**c**). Data are presented as mean ± standard deviation (*n* = 5). Lowercase letters in the histograms indicate statistical differences between groups (*p* < 0.05).

Mitochondrial ROS levels were increased in the Aβ group (136.98%) compared to the NC group (100.00%) (Figure 3a). However, the mitochondrial ROS levels were decreased in the MG4247 and MG4644 (106.88% and 120.59%, respectively) groups compared to the Aβ group.

MMP was reduced in the Aβ group (39.58%) compared to the NC group (100.00%) (Figure 3b). However, the MMP was enhanced in the MG4247 and MG4644 (65.89% and 72.03%, respectively) groups compared to the Aβ group.

Mitochondrial ATP contents were decreased in the Aβ group (15.14 nmole/mg of protein) compared to the NC group (20.91 nmole/mg of protein) (Figure 3c). However, the mitochondrial ATP contents were increased in the MG4247 and MG4644 (19.71 and 19.77 nmole/mg of protein, respectively) groups compared to the Aβ group.

### 3.4. L. gasseri MG4247 and L. rhamnosus MG4644 Enhance Synaptic Plasticity

**Figure 4:**
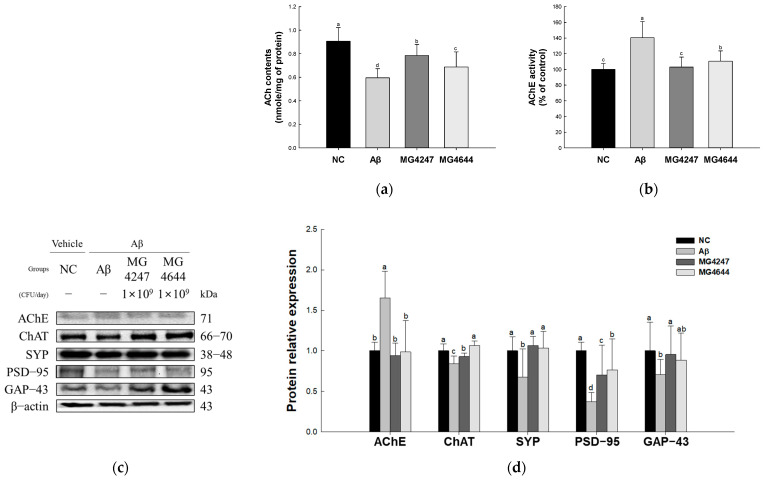
Effects of *L. gasseri* MG4247 and *L. rhamnosus* MG4644 on synaptic plasticity of brain tissue in Aβ-induced cognitive dysfunction mice. Acetylcholine (ACh) contents (**a**) and acetylcholinesterase (AChE) activity (**b**); these data are shown as mean ± standard deviation (*n* = 5). Western blot band images (**c**) and protein expression levels of AChE, ChAT, SYP, PSD-95, and GAP-43 (**d**), and these data are shown as mean ± standard deviation (*n* = 3). Lowercase letters in the histograms indicate statistical differences between groups (*p* < 0.05).

ACh contents were reduced in the Aβ group (0.60 nmole/mg of protein) compared to the NC group (0.91 nmole/mg of protein) (Figure 4a). However, the ACh contents were enhanced in the MG4247 and MG4644 (0.78 and 0.69 nmole/mg of protein, respectively) groups compared to the Aβ group.

AChE activity was increased in the Aβ group (140.38%) compared to the NC group (100.00%) (Figure 4b). However, AChE activity was decreased in the MG4247 and MG4644 (103.07% and 110.58%, respectively) groups compared to the Aβ group.

The expression levels of AChE increased in the Aβ group (1.66) compared to the NC group (1.00). The expression levels of ChAT, SYP, PSD-95, and GAP-43 decreased in the Aβ group (0.84, 0.67, 0.37, and 0.71, respectively) compared to the NC group (1.00). However, the expression levels of AChE, ChAT, SYP, PSD-95, and GAP-43 were improved in the MG4247 group (0.94, 0.93, 1.06, 0.70, and 0.95, respectively) and the MG4644 group (0.98, 1.06, 1.06, 0.77, and 0.88, respectively) compared to the Aβ group.

### 3.5. L. gasseri MG4247 and L. rhamnosus MG4644 Strengthen the BBB by Modulating Expression Levels of Tight Junction (TJ) Proteins

**Figure 5:**
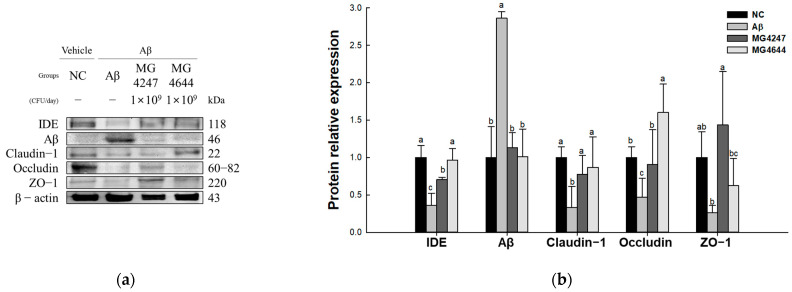
Effects of *L. gasseri* MG4247 and *L. rhamnosus* MG4644 on tight junctions of brain tissue in Aβ-induced cognitive dysfunction mice. Western blot band images (**a**) and protein expression levels of IDE, claudin-1, occludin, ZO-1, and Aβ (**b**). Data are presented as mean ± standard deviation (*n* = 3). Lowercase letters in the histograms indicate significant statistical differences between groups (*p* < 0.05).

To confirm the strength of the BBB effect of *L. gasseri* MG4247 and *L. rhamnosus* MG4644 on Aβ-induced cognitive impairment, the expression levels of TJ-related proteins were measured (Figure 5). The expression levels of Aβ (2.86) increased in the Aβ group compared to the NC group (1.00). The expression levels of IDE (0.36), claudin-1 (0.33), occludin (0.47), and ZO-1 (0.26) decreased in the Aβ group compared to the NC group (1.00). However, the MG4247 and MG4644 groups had improved expression levels of IDE (0.70 and 0.97), Aβ (1.13 and 1.01), claudin-1 (0.77 and 0.87), occludin (0.90 and 1.60), and ZO-1 (1.44 and 0.63) compared to the Aβ group.

### 3.6. L. gasseri MG4247 and L. rhamnosus MG4644 Suppress Neuroinflammation Through Inhibition of the TLR4 Pathway

**Figure 6:**
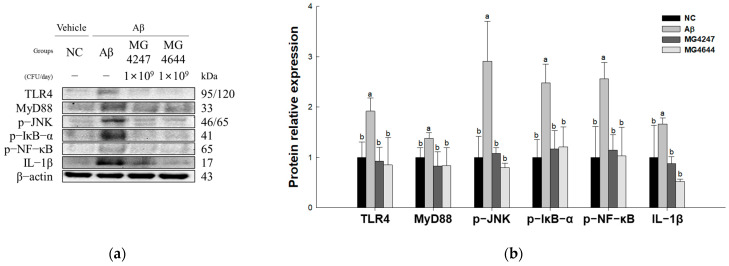
Effects of *L. gasseri* MG4247 and *L. rhamnosus* MG4644 on neuroinflammation of brain tissue in Aβ-induced cognitive dysfunction mice. Western blot band images (**a**) and protein expression levels of TLR4, MyD88, p-JNK, p-IκB-α, p-NF-κB, and IL-1β (**b**). Data are presented as mean ± standard deviation (*n* = 3). Lowercase letters in the histograms indicate statistical differences between groups (*p* < 0.05).

To confirm the neuroinflammation-ameliorating effect of *L. gasseri* MG4247 and *L. rhamnosus* MG4644 on Aβ-induced cognitive impairment, the expression levels of TLR4 signaling pathway proteins were measured (Figure 6). The expression levels of TLR4 (1.92), MyD88 (1.38), p-JNK (2.91), p-IκB-α (2.48), p-NF-κB (2.56), and IL-1β (1.66) increased in the Aβ group compared to the NC group (1.00). However, the MG4247 and MG4644 groups had improved expression levels of TLR4 (0.92 and 0.8), MyD88 (0.83 and 0.8), p-JNK (1.08 and 0.8), p-IκB-α (1.17 and 1.2), p-NF-κB (1.14 and 1.03), and IL-1β (0.87 and 0.52) compared to the Aβ group.

### 3.7. L. gasseri MG4247 and L. rhamnosus MG4644 Attenuate Neurotoxicity Through Regulation of the Akt Pathway

**Figure 7:**
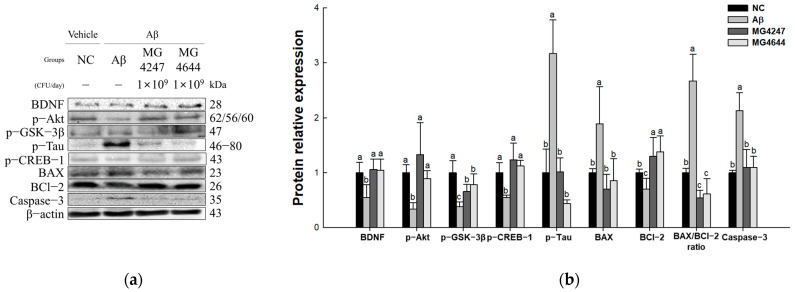
Effects of *L. gasseri* MG4247 and *L. rhamnosus* MG4644 on neurotoxicity of brain tissue in Aβ-induced cognitive dysfunction mice. Western blot band images (**a**) and protein expression levels of BDNF, p-Akt, p-GSK-3β, p-tau, p-CREB-1, BAX, BCl-2, BAX/BCl-2 ratio, and caspase-3 (**b**). Data are presented as mean ± standard deviation (*n* = 3). Lowercase letters in the histograms indicate statistical differences between groups (*p* < 0.05).

The expression levels of Akt signaling pathway proteins were measured to confirm the neurotoxicity-ameliorating effect of *L. gasseri* MG4247 and *L. rhamnosus* MG4644 on Aβ-induced cognitive impairment (Figure 7). The expression levels of p-tau, BAX, BAX/BCl-2 ratio, and caspase-3 increased in the Aβ group (3.17, 1.89, 2.67, and 2.13, respectively) compared to the NC group (1.00). The expression levels of BDNF, p-Akt, p-GSK-3β, p-CREB-1, and BCl-2 decreased in the Aβ group (0.55, 0.34, 0.37, 0.54, and 0.70, respectively) compared to the NC group. However, the expression levels of BDNF, p-Akt, p-GSK-3β, p-tau, p-CREB-1, BAX, BCl-2, BAX/BCl-2 ratio, and caspase-3 were improved in the MG4247 group (1.06, 1.33, 0.66, 1.01, 1.23, 0.71, 1.30, 0.54, and 1.10, respectively) and the MG4644 group (1.05, 0.89, 0.78, 0.44, 1.13, 0.85, 1.38, 0.61, and 1.10, respectively) compared to the Aβ group.

### 3.8. L. gasseri MG4247 and L. rhamnosus MG4644 Improve Dysbiosis

**Figure 8:**
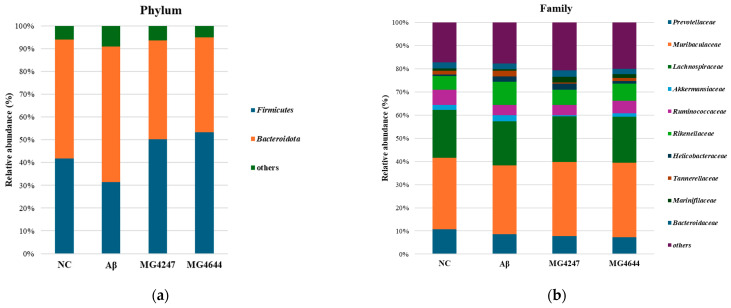
Effects of *L. gasseri* MG4247 and *L. rhamnosus* MG4644 on gut microbiome composition. The relative abundance (%) of the phylum (**a**) and family (**b**) levels in each group. Data are presented as mean ± standard deviation (*n* = 3).

To determine the effects of the ingestion of *L. gasseri* MG4247 and *L. rhamnosus* MG4644 on the GMB, we measured the relative abundance of bacteria (Figure 8). Table 1 shows the overall change patterns of the top 10% most abundant bacteria in mouse feces.

The analysis results are summarized in Table 1, presenting taxa with high relative abundances at the phylum, family, and genus levels. In the Aβ group, the relative abundances of *Firmicutes*, the *Firmicutes/Bacteroidota* ratio, *Lachnospiraceae*, and *Lachnospiraceae* NK4A136 were reduced compared to the NC group. However, in the MG4247 and MG4644 groups, these abundances increased relative to the Aβ group. On the other hand, the relative abundances of *Bacteroidota*, *Rikenellaceae*, *Tannerellaceae*, *Helicobacter*, *Rikenellaceae* RC9, *Bacteroides*, and *Alistipes* were elevated in the Aβ group compared to the NC group. In contrast, these abundances decreased in the MG4247 and MG4644 groups compared to the Aβ group.

**Figure 9:**
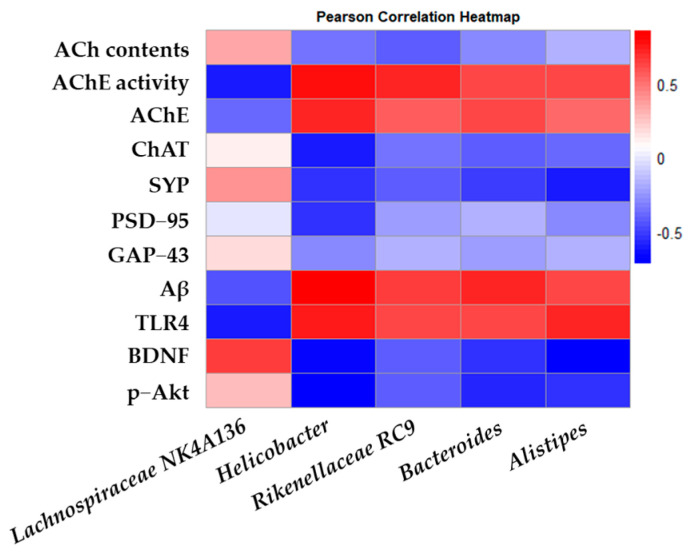
Effects of *L. gasseri* MG4247 and *L. rhamnosus* MG4644 on correlation analysis. Heatmap of Pearson correlations between gut microbiota genus level and key factors related to cognitive function.

The Pearson correlation between the intestinal microbiota and biochemical indices in the mouse model was analyzed and represented as a heatmap (Figure 9). Through correlation analysis, *Helicobacter*, *Rikenellaceae* RC9, *Bacteroides*, and *Alistipes*, known as harmful bacteria, showed a positive correlation with AChE activity, AChE, Aβ, and TLR4, and *Lachnospiraceae* NK4A136, known as beneficial bacteria, showed a positive correlation with ACh contents and expressions of ChAT, SYP, GAP-43, BDNF, and p-Akt.

## 4. Discussion

AD is the most common neurodegenerative disease found in the elderly, and it is characterized by clinical symptoms such as progressive memory loss, cognitive decline, and daily life dysfunction [1]. The accumulation of Aβ, one of the main pathological features of AD, causes oxidative stress, which induces mitochondrial dysfunction, neuroinflammation, and neurotoxicity, thereby promoting neuroapoptosis in brain tissue [4]. Neuroapoptosis ultimately contributes to cognitive dysfunctions such as reduced memory and learning abilities, which are characteristic of AD [11]. Research on resources that can effectively protect against this is ongoing. In this regard, as research results have recently been published showing that LAB can improve cognitive dysfunction by alleviating GMB imbalance, it is attracting attention as a material with new neuroprotective effects [14]. In particular, *L. gasseri* and *L. rhamnosus*, which correspond to LAB, are generally recognized as safe (GRAS) strains and their safety and beneficial effects have been proven [25,26]. Therefore, this study investigated the neuroprotective effect of the intake of *L. gasseri* MG4247 and *L. rhamnosus* MG4644 in an Aβ-induced cognitive impairment mouse model.

The main pathological feature of AD is the accumulation of Aβ peptides consisting of 40–42 amino acids, which are produced by enzymatic cleavage of the amyloid precursor protein outside the cell [27]. This accumulation of Aβ stimulates the activation of glial cells and causes oxidative stress and neurotoxicity, which in turn leads to persistent damage to neurons and ultimately to cognitive and memory loss [28]. Memory loss can be assessed by behavioral tests such as the Y-maze, passive avoidance, and MWM in AD animal models to confirm changes in long-term and short-term memory [29]. This is considered an important initial step to confirm the potential of learning and memory effects and conduct mechanism studies [29]. Therefore, we aimed to evaluate the learning and memory effects of *L. gasseri* MG4247 and *L. rhamnosus* MG4644. As a result, the Aβ group had increased working memory (Y-maze test), long-term and short-term memory for fear stimuli (passive avoidance test), spatial cognitive ability, and long-term memory (MWM test), establishing an Aβ-induced cognitive decline model (Figure 1). In a previous study, it was reported that *L. gasseri* NK109 intake changed the composition of GMB in adults with mild cognitive impairment and alleviated cognitive dysfunction in the Y-maze and Barnes maze tests [30]. In addition, it was reported that *L. rhamnosus* intake showed a protective effect in the MWM and Y-maze tests with the regulation of GMB in a memory deficit model induced by lead acetate exposure [31]. Consistent with these previous findings, our results confirmed that oral administration of *L. gasseri* MG4247 and *L. rhamnosus* MG4644 had anti-neurodegenerative effects in a model of Aβ-induced cognitive deficits. Further studies were conducted to elucidate the mechanisms underlying these effects.

Aβ peptides that have accumulated exhibit a strong affinity for redox-active metal ions like iron (Fe^2+^/Fe^3+^), promoting the Fenton reaction and contributing to oxidative stress by producing ROS [4]. Brain tissue has a relatively low antioxidant capacity and is particularly susceptible to oxidative stress due to its high oxygen consumption and abundant unsaturated fatty acids [28,32]. This oxidative stress causes excessive lipid peroxide accumulation from lipid peroxidation, which can lead to neuroapoptosis and cognitive dysfunction [28,32]. Oxidative stress not only damages lipids, proteins, and DNA in cell membranes, but also particularly inhibits the structural and functional plasticity of synapses [5]. To protect against this, antioxidant enzymes such as SOD, catalase (CAT), and glutathione peroxidase (GPx) exist in the body, which remove ROS or suppress damage [31]. However, excessive accumulation of oxidative stress can damage the antioxidant system and cause neuronal death, leading to cognitive dysfunction [28]. Therefore, we evaluated the effects of *L. gasseri* MG4247 and *L. rhamnosus* MG4644 intake on the antioxidant system. As a result, we confirmed that the SOD contents and GSH levels decreased and the MDA contents increased in the Aβ group (Figure 2). In a previous study, *L. gasseri* SBT2055 intake showed an improvement effect on oxidative stress by upregulating the relative expression levels of SOD and heme oxygenase (HO-1) [33]. In addition, *L. rhamnosus* GG intake showed an effect of improving antioxidant capacity by upregulating the levels of antioxidant enzymes CAT and GPx in a liver injury mouse model [34]. Consistent with these previous study results, our study results suggest that oral administration of *L. gasseri* MG4247 and *L. rhamnosus* MG4644 inhibited the accumulation of MDA in brain tissue by protecting antioxidant enzymes such as SOD and GSH. Therefore, these results suggest that *L. gasseri* MG4247 and *L. rhamnosus* MG4644 intake can improve the antioxidant system by reducing Aβ-induced ROS accumulation, thereby contributing to the prevention of cognitive dysfunction.

Excessive ROS generated by Aβ causes the loss of mitochondrial membrane potential, which disrupts the function of the electron transport chain [5]. This causes the movement of electrons and ions essential for energy production within the mitochondria to be impaired, resulting in decreased ATP production and failure to meet the ATP demand of neurons [5,35]. Mitochondria not only play a central role in intracellular energy production, but also play an important role in maintaining calcium homeostasis and regulating cell survival signals [11]. However, when ROS accumulates, it causes mitochondrial DNA damage and membrane protein oxidation, further aggravating mitochondrial dysfunction [36]. This mitochondrial dysfunction impairs synaptic plasticity and signal transmission between pre- and post-synaptic neurons, which are important for learning and memory, by failing to meet the energy demand of synapses [5]. In this regard, it has been reported that short-chain fatty acids produced by beneficial bacteria in the GMB can contribute to improving mitochondrial dysfunction by promoting mitochondrial energy production, increasing ATP synthesis, and reducing oxidative stress [37]. To confirm this, this study evaluated the effects of the consumption of *L. gasseri* MG4247 and *L. rhamnosus* MG4644 on Aβ-induced mitochondrial dysfunction. As a result, the Aβ group showed an increase in ROS levels and decreased MMP levels and ATP contents (Figure 3). A previous study reported that *Lacticaseibacillus paracasei* PS23 showed an effect of delaying muscle loss due to aging by decreasing ROS levels and maintaining MMP and ATP levels in an aging mouse model [35]. In addition, *L. rhamnosus* Fmb14 was shown to decrease mitochondrial ROS levels and restore MMP in uric-acid-induced HepG2 cells [36]. Consistent with previous research results, the present study confirmed that oral administration of *L. gasseri* MG4247 and *L. rhamnosus* MG4644 effectively removed ROS by protecting the antioxidant system, thereby reducing ROS content in mitochondria. This decrease in ROS content is thought to improve mitochondrial dysfunction by maintaining mitochondrial membrane potential and promoting ATP synthesis. Therefore, these results suggest that *L. gasseri* MG4247 and *L. rhamnosus* MG4644 intake can reduce Aβ-induced mitochondrial dysfunction and prevent cognitive dysfunction as a result.

Aβ oligomers bind to presynaptic and postsynaptic membranes and inhibit the expression of proteins essential for synaptic function and stability [5]. Specifically, Aβ decreases the levels of SYP, a presynaptic vesicle protein important for neurotransmitter release, and PSD-95, which plays a key role in maintaining synaptic structure and function [6]. In addition, Aβ decreases the expression of GAP-43, which is involved in axonal growth, synaptic remodeling, and neural network stabilization [38]. This reduces synapse density, causes structural degeneration of dendritic spines, weakens synaptic functional connectivity, and inhibits synaptic plasticity, the neural basis of learning and memory [6]. In particular, Aβ impairs the reorganization and connection strength of neural networks by inhibiting long-term potentiation (LTP) and excessively inducing long-term weakening (LTD) [5]. These changes reduce signal transmission efficiency between neurons and impair memory and learning ability [5,6]. ACh is a neurotransmitter associated with synaptic plasticity and plays an important role in learning and memory processes [38]. ACh is produced by the combination of acetyl-CoA and choline by ChAT, stored in presynaptic vesicles, and released into the synaptic gap during neural signal transmission [39]. The released ACh binds to receptors located in the postsynaptic membrane, transmits signals, and is then degraded and recycled by AChE [39]. However, Aβ induces overexpression of AChE, which excessively promotes the degradation of ACh in the synaptic cleft [38]. This causes a rapid decrease in the concentration of ACh, which reduces neurotransmission efficiency and causes dysfunction of the cholinergic system [40]. According to recent studies, GMB affects synaptic plasticity and neurotransmitter regulation through the gut–brain axis, and *Lactobacillus* can contribute to upregulating the expression of synapse-related proteins [15]. To confirm this, this study evaluated the effect of the consumption of *L. gasseri* MG4247 and *L. rhamnosus* MG4644 on Aβ-induced synaptic plasticity. As a result, in the Aβ group, the cholinergic transmission process was confirmed to be deficient due to a decrease in ACh content and an increase in AChE activity, and the expression level of synapse-related proteins was also weakened (Figure 4). In a previous study, it was reported that *L. gasseri* NK109 intake contributed to the improvement of cognitive function by increasing the expression of BDNF and p-CREB in aged mice [30]. In addition, it was reported that *L. rhamnosus* GG intake improved cognitive and memory impairment by increasing the expression of SYP, PSD-95, and BDNF in an ethanol-exposed mouse model [41]. These results suggest that the intake of *L gasseri* MG4247 and *L. rhamnosus* MG4644 can contribute to improving Aβ-induced cognitive dysfunction by protecting synaptic plasticity.

Accumulation of Aβ induces the production of ROS and secretion of inflammatory cytokines, thereby compromising BBB integrity by downregulating the expression of TJ proteins occludin, claudin-1, and ZO-1 [6,15,42]. In fact, it has been reported that BBB permeability increased as the expression of TJ proteins claudin-5 and ZO-1 decreased in an Alzheimer’s model [43]. The BBB is a physical and metabolic barrier composed of endothelial cells, basement membrane, and TJ proteins, and its role is to block the entry of toxic substances, pathogens, and inflammatory factors into the brain and maintain brain homeostasis [44]. Claudin-1 regulates the movement of selective ions and molecules, and is a key protein in the TJ structure, stabilizing the connection of endothelial cells [44]. Occludin serves as a physical barrier for the BBB by strengthening adhesion between cells and is also involved in cell signaling [44]. ZO-1 connects claudin-1 and occludin to the cytoskeleton, thereby systematically stabilizing the TJ complex and regulating signaling pathways [42]. In the pathological damage process caused by Aβ, IDE plays an important role in preventing TJ protein damage and maintaining BBB integrity by efficiently degrading Aβ [45]. However, in AD patients, IDE activity is reduced, which reduces Aβ degradation and further aggravates BBB damage and TJ protein expression reduction [45]. To confirm this, this study evaluated the effects of *L. gasseri* MG4247 and *L. rhamnosus* MG4644 intake on BBB integrity. As a result, the Aβ group showed a decrease in the expression level of TJ-related proteins (Figure 5). In a previous study, *L. gasseri* JM1 showed the effect of alleviating the damaged barrier structure by increasing the expression of claudin-3, occludin, and ZO-1 and regulating inflammatory and anti-inflammatory cytokines in a mouse model of colitis [46]. In addition, exopolysaccharides derived from *L. rhamnosus* were reported to reduce brain damage by promoting the expression of occludin, claudin-1, and ZO-1 in the brain tissue of a D-galactose-induced mouse model and effectively reducing the expression of inflammatory cytokines [42]. Consistent with previous findings, the present study suggests that oral administration of *L. gasseri* MG4247 and *L. rhamnosus* MG4644 increased IDE expression, which enhanced the degradation of Aβ and improved the expressions of TJ proteins such as occludin, claudin-1, and ZO-1. Therefore, these results suggest that *L. gasseri* MG4247 and *L. rhamnosus* MG4644 intake may contribute to alleviating Aβ-induced BBB damage and preventing cognitive dysfunction.

Neuroinflammation refers to a persistent inflammatory condition in the CNS driven by the activation of microglia and astrocytes in response to Aβ plaques, which subsequently produce a substantial number of inflammatory cytokines [47]. This inflammatory response can reduce synaptic plasticity, cause neuronal dysfunction, and ultimately lead to cognitive dysfunction [6]. In particular, according to a study by XIN et al., synaptic plasticity was reduced, inflammatory cytokines were upregulated, and memory impairment occurred in a mouse model in which hyperimmune responses were induced by repeated intraperitoneal injection of lipopolysaccharide (LPS) [48]. Microglia are the main immune cells responsible for innate immunity in the CNS and are involved in the early defense mechanism that rapidly responds to external pathogens or internal damage signals and regulates neuroinflammatory responses [9]. The core of this early defense mechanism is the expression of pattern recognition receptors (PRRs), which detect pathogen-associated molecular patterns (PAMPs) or damage-associated molecular patterns (DAMPs) to induce immune responses [49]. Among them, TLR4 is expressed on the surface of microglia and is activated by recognizing Aβ as a DAMP when Aβ accumulates in the CNS [7]. TLR4 forms a complex with myeloid differentiation factor 2 (MD2) accessory protein binding to Aβ and forming dimerization through structural changes after ligand binding [49]. This dimerization of TLR4 activates the IκB-α, NF-κB, and JNK pathways via the MyD88 adaptor protein, ultimately inducing the expression of inflammatory cytokines such as IL-1β [34]. Excessive activation of these pathways aggravates neuroinflammation and further weakens synaptic plasticity by abnormally regulating the synaptic pruning process [50]. Therefore, we measured the effects of *L. gasseri* MG4247 and *L. rhamnosus* MG4644 intake on TLR4 pathway activation. As a result, the Aβ group showed an increase in the expression level of proteins related to the TLR4 signaling pathway (Figure 6). In a previous study, *L. gasseri* JM1 intake suppressed the expression of inflammatory cytokines such as TNF-α and increased the expression of anti-inflammatory cytokines such as IL-10, thereby alleviating intestinal inflammation in a mouse model of colitis [46]. In addition, *L. gasseri* NK109 showed a protective effect that alleviated neuroinflammation and cognitive impairment by decreasing the expression of IL-1β and increasing the expression of the anti-inflammatory cytokine IL-10 [30]. Also, *L. rhamnosus* GG showed a hepatoprotective effect by regulation of the TLR4/NF-κB pathway, reducing the expression of IL-1β and TNF-α in a liver injury mouse model [34]. Consistent with previous findings, the present study confirmed that oral administration of *L. gasseri* MG4247 and *L. rhamnosus* MG4644 decreased TLR4 expression by regulating microglial activation involved in neuroinflammation. This is thought to have reduced IL-1β expression by regulating NF-κB signaling, a downstream inflammatory response of TLR4. Therefore, these results suggest that intake of *L. gasseri* MG4247 and *L. rhamnosus* MG4644 can alleviate neuroinflammation by inhibiting the Aβ-induced TLR4/NF-κB pathway, thereby improving synaptic plasticity and cognitive function.

Aβ activates the NF-κB pathway with neuroinflammatory response and activated NF-κB inhibits BDNF expression, reducing neuronal survival and synaptic plasticity [30] as a result. BDNF is an essential neurotrophic factor that regulates the survival, growth, and synaptic plasticity of neurons in the CNS, and plays a key role in memory and learning [51]. The expression of BDNF is regulated by CREB, and when neurons are exposed to BDNF, CREB is phosphorylated through the mitogen-activated protein kinase (MAPK) pathway [52]. Phosphorylated CREB binds to the promoter of the BDNF gene and induces BDNF transcription, which plays an important role in maintaining neuronal survival and synaptic function [52]. Following phosphorylation of CREB, activation of BDNF activates the Akt/GSK-3β pathway through the tropomyosin receptor kinase B (TrkB) receptor [9]. Akt is a key signaling protein that regulates cell survival and death, and when activated, it inhibits the activity of GSK-3β and blocks apoptosis signals [9]. However, when BDNF expression and the Akt pathway are inhibited by Aβ, GSK-3β is abnormally activated, inducing hyperphosphorylation of tau, which accelerates tau accumulation, a major pathological feature of AD [52]. Tau accumulation disrupts the BAX/BCl-2 balance, activating caspase-3 and producing neuronal apoptosis [10]. Accordingly, considering that the Akt pathway plays an important role in neuronal viability and neuroapoptosis regulation, we evaluated the effects of *L. gasseri* MG4247 and *L. rhamnosus* MG4644 consumption on Akt pathway activity. As a result, the expression of BDNF decreased in the Aβ group, inhibiting the phosphorylation of the Akt signaling pathway and increasing the expression level of proteins related to neuroapoptosis (Figure 7). In a previous study, it was reported that the consumption of *Lactobacillus spp.* has a protective effect in gamma-irradiated mice by regulating the apoptosis-related genes BCl-2, BAX, and caspase-3 [53]. In addition, *L. rhamnosus* GG was shown to improve cognitive–behavioral deficits in a sepsis mouse model by regulating BDNF expression and p-TrkB levels [54]. Consistent with previous findings, we confirmed in this study that oral administration of *L. gasseri* MG4247 and *L. rhamnosus* MG4644 increased the expression of BDNF, which plays an important role in regulating neuronal survival and synaptic plasticity and activated the Akt signaling pathway through this. Activation of the Akt pathway increased the expression of CREB, which is related to neuronal survival while decreasing the expression of proteins such as tau, BAX, and caspase-3, which are involved in neuronal apoptosis. These results suggest that the intake of *L. gasseri* MG4247 and *L. rhamnosus* MG4644 may improve synaptic plasticity by regulating the Akt signaling pathway with BDNF expression.

Accumulation of Aβ increases ROS production and the secretion of pro-inflammatory cytokines, which accelerates the breakdown of the BBB [12]. As a result, ROS and inflammatory cytokines generated by Aβ can enter the bloodstream and reach the gut, where they enhance intestinal permeability, leading to GMB dysbiosis [12]. A notable example of GMB imbalance observed in AD models is the reduction in *Firmicutes* and the increase in *Bacteroidota* [55]. *Bacteroidota* is a Gram-negative bacterium, and its outer membrane containing LPS is known to activate the TLR4 pathway and aggravate inflammatory responses and AD pathology [55]. Moreover, pathogenic bacteria such as *Escherichia coli* (*E. coli*) increase under GMB imbalance and release bacterial-derived LPS and amyloids, such as curli fibers, into the intestine through metabolic processes [56]. These bacterial amyloids have structural properties similar to human amyloid beta and induce the production of inflammatory cytokines by activating the TLR4/NF-κB pathway [12]. Metabolic byproducts such as LPS and inflammatory cytokines formed in the gut can re-enter the bloodstream and travel to the brain, further compromising BBB integrity and triggering inflammatory responses, and accelerating AD progression as a result [13]. Therefore, we evaluated the effects of the intake of *L. gasseri* MG4247 and *L. rhamnosus* MG4644, known as dietary LAB, on GMB imbalance. As a result, the MG4247 and MG4644 groups restored the levels of *Lachnospiraceae* NK4A136, which plays an important role in improving cognition [57], while reducing the levels of *Helicobacter*, *Rikenellaceae* RC9, and *Bacteroides*, which are known as harmful bacteria [58,59], thereby protecting the intestinal mucosa layer (Figure 8, Table 1). According to a previous study, it was reported that the proportion of *Helicobacter*, *Rikenellaceae* RC9, and *Bacteroides*, which are related to cognitive decline, increased in AD mouse models [58,59]. In addition, *Lachnospiraceae* NK4A136 showed a negative correlation with the risk of vascular dementia, which was reported to have a protective effect against vascular dementia [57]. Moreover, in our previous study using a DSS-induced colitis mouse model, dietary supplementation with LAB significantly increased the abundance of *Firmicutes* and *Lachnospiraceae* NK4A136, which increased SCFA production, contributing to improved cognitive function [24]. Similarly, our current results showed similar changes in gut microbiota, suggesting that changes in gut microbiota composition may affect cognitive function via metabolic regulation. In this study, we performed a correlation analysis between gut microbiota and the major cognitive-function-related biomarkers and biochemical indices. As a result, our analysis showed that *Lachnospiraceae* NK4A136 had a positive correlation with cognitive function protection indicators, whereas *Helicobacter*, *Rikenellaceae* RC9, and *Bacteroides* had a negative correlation (Figure 9). Yun et al. reported that oral administration of *L. gasseri* NK109 decreased the abundance of *Helicobacter* in a cognitive impairment mouse model [30]. In addition, oral administration of *L. rhamnosus* GR-1 in a mouse model of lead-induced cognitive impairment was shown to reorganize the gut microbiota by enhancing the proportion of *Firmicutes* and improving memory deficits [60]. Also, the administration of exopolysaccharides derived from *L. rhamnosus* was reported to increase the level of the *Lachnospiraceae* family and decrease the level of *Helicobacter* in a D-galactose-induced inflammatory brain injury model [42]. Therefore, these results suggest that the intake of *L. gasseri* MG4247 and *L. rhamnosus* MG4644 can help improve GMB dysbiosis by increasing beneficial intestinal bacteria and reducing harmful bacteria. In addition, probiotics are known to affect the composition of the gut microbiota [20], and long-term intake of these probiotics may enhance cognitive improvement effects through the gut–brain axis due to changes in metabolites such as SCFAs and neurotransmitters as a result of the increase in beneficial gut bacteria over time. To confirm this hypothesis, future studies are needed to investigate the changes in the gut microbiota according to the duration of probiotic intake, the safety assessment of long-term intake of newly identified strains, metabolomic analysis, and the mechanism of the gut–brain axis connection. Nevertheless, this study showed that both probiotics may be used as a potential preventive strategy to improve Aβ-induced cognitive impairment.

## 5. Conclusions

This study evaluated the protective effects of two LAB strains (*L. gasseri* MG4247 and *L. rhamnosus* MG4644) selected through preliminary studies in a model of cognitive dysfunction induced by Aβ and their industrial applicability as functional foods. Oral administration of *L. gasseri* MG4247 and *L. rhamnosus* MG4644 maintained the antioxidant system, improved mitochondrial function and the expression of synaptic-plasticity-related proteins, and enhanced BBB integrity by increasing the expression of TJ proteins. In addition, the two strains effectively alleviated neuroinflammation and neurotoxicity by regulating the TLR4/Akt signaling pathway. This protective effect is believed to have contributed to alleviating mitochondrial dysfunction and BBB damage by reducing Aβ-induced oxidative stress, preventing neuroinflammation and neuronal death, restoring synaptic plasticity, and improving cognitive function. In conclusion, this study suggests that *L. gasseri* MG4247 and *L. rhamnosus* MG4644 may protect cognitive function by inducing changes in the GMB and regulating the TLR4/Akt signaling pathway to enhance synaptic plasticity. However, additional research is needed to determine their direct effects on the TLR4/Akt pathway so that these strains can be utilized industrially as probiotic-based health functional food materials.

## Figures and Tables

**Table 1:** Effects of *L. gasseri* MG4247 and *L. rhamnosus* MG4644 on the relative abundances of gut microbiome composition in Aβ-induced cognitive dysfunction mice.

Level		NC	Aβ	MG4247	MG4644
Phylum	*Firmicutes*	43.62 ± 8.32 ^b^	29.60 ± 1.46 ^c^	55.39 ± 13.27 ^a^	60.10 ± 10.98 ^a^
	*Bacteroidota*	49.56 ± 9.55 ^ab^	62.20 ± 2.08 ^a^	37.22 ± 16.35 ^b^	35.84 ± 12.47 ^b^
	*Firmicutes*/*Bacteroidota* ratio	85.26 ± 9.33 ^b^	54.23 ± 3.62 ^c^	115.18 ± 15.56 ^a^	120.68 ± 10.38 ^a^
Family	*Lachnospiraceae*	31.93 ± 17.78 ^b^	12.76 ± 4.05 ^c^	39.41 ± 12.25 ^ab^	50.35 ± 12.26 ^a^
	*Rikenellaceae*	7.77 ± 1.41 ^b^	12.74 ± 4.20 ^a^	7.09 ± 2.78 ^b^	8.21 ± 1.54 ^b^
	*Tannerellaceae*	2.21 ± 0.84 ^ab^	3.01 ± 1.15 ^a^	0.61 ± 0.07 ^c^	1.54 ± 1.03 ^bc^
Genus	*Lachnospiraceae NK4A136*	0.12 ± 0.05 ^ab^	0.04 ± 0.01 ^b^	0.22 ± 0.13 ^a^	0.16 ± 0.05 ^ab^
	*Helicobacter*	0.01 ± 0.00 ^c^	0.03 ± 0.00 ^a^	0.01 ± 0.00 ^b^	0.01 ± 0.00 ^c^
	*Rikenellaceae RC9*	0.02 ± 0.01 ^b^	0.05 ± 0.01 ^a^	0.01 ± 0.00 ^b^	0.02 ± 0.01 ^b^
	*Bacteroides*	0.03 ± 0.00 ^b^	0.05 ± 0.01 ^a^	0.01 ± 0.00 ^d^	0.02 ± 0.00 ^c^
	*Alistipes*	0.04 ± 0.01 ^b^	0.06 ± 0.02 ^a^	0.04 ± 0.02 ^b^	0.03 ± 0.01 ^b^

Data are presented as mean ± standard deviation (*n* = 3). Lowercase letters in each row of the table indicate significant statistical differences between groups (*p* < 0.05).

## Data Availability

The data presented in this study are available on request from the corresponding author.

## Supplementary Materials

The following supporting information can be downloaded at: https://www.mdpi.com/article/10.3390/antiox14020139/s1, Figure S1: Neuroprotective effects of cell-free supernatants (CFSs) in HT22 and SK-N-MC cells; Table S1: The isolates and accession numbers of lactic acid bacteria (LAB) strains used in this study; Table S2: List of primary and secondary antibodies information used in this study.
