# Supplementary material for: Oral Administration of Lactobacillus gasseri and Lacticaseibacillus rhamnosus Ameliorates Amyloid Beta (Aβ)-Induced Cognitive Impairment by Improving Synaptic Function Through Regulation of TLR4/Akt Pathway
Source: Antioxidants (Basel). 2025 Jan 24;14(2):139. doi: 10.3390/antiox14020139 (PMC11851505; doi:10.3390/antiox14020139)
Supplement: Supplementary file 1 [file antioxidants-14-00139-s001.zip › antioxidants-3433315-supplementary.pdf]

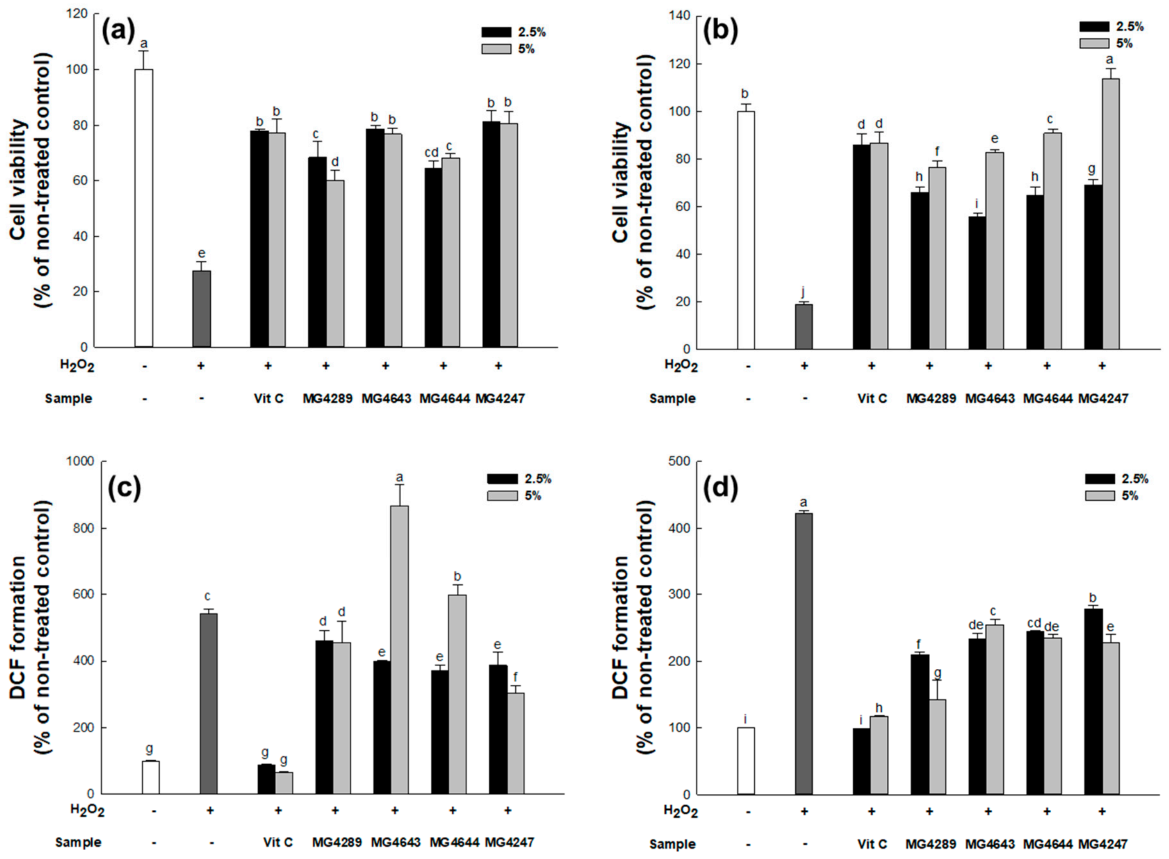

Figure S1. Neuroprotective effects of 2.5% and 5% cell-free supernatants (CFS) in HT22 and SK-N-MC cells. The cell viability (**a** and **b**) and intracellular reactive oxygen species (ROS) level (**c** and **d**) in H_2O_2-treated HT22 and SK-N-MC cells. The results shown were mean ± standard deviation ($n = 3$). Lowercase letters in the histogram indicate statistical differences between groups ($p < 0.05$). MG4289, *Lacticaseibacillus rhamnosus* MG4289; MG4643, *Lacticaseibacillus rhamnosus* MG4643; MG4644, *Lacticaseibacillus rhamnosus* MG4644; and MG4247, *Lactobacillus gasseri* MG4247.

Table S1. The isolates and accession numbers of lactic acid bacteria (LAB) strains used in this study.

Origin	LAB	NCBI accession number
Human	*Lactobacillus gasseri* MG4247	MN069036.1
	Lacticaseibacillus rhamnosus MG4644	ON668170.1

NCBI, National Center for Biotechnology Information

Table S2. List of primary and secondary antibodies information used in this study.

Antibody	Catalog NO.	Manufacturer
β-actin	sc-69879	Santa Cruz Biotechnology (Dallas, TX, USA)
AChE	sc-373901	Santa Cruz Biotechnology (Dallas, TX, USA)
ChAT	20747-1-AP	Proteintech (Rosemont, IL, USA)
SYP	sc-17750	Santa Cruz Biotechnology (Dallas, TX, USA)
PSD-95	sc-32290	Santa Cruz Biotechnology (Dallas, TX, USA)
GAP-43	sc-17790	Santa Cruz Biotechnology (Dallas, TX, USA)
IDE	sc-393887	Santa Cruz Biotechnology (Dallas, TX, USA)
Claudin-1	sc-166338	Santa Cruz Biotechnology (Dallas, TX, USA)
Occludin	sc-133256	Santa Cruz Biotechnology (Dallas, TX, USA)
ZO-1	sc-33725	Santa Cruz Biotechnology (Dallas, TX, USA)
TLR4	sc-293072	Santa Cruz Biotechnology (Dallas, TX, USA)
MyD88	sc-74532	Santa Cruz Biotechnology (Dallas, TX, USA)
p-JNK	sc-6254	Santa Cruz Biotechnology (Dallas, TX, USA)
p-IκB-α	sc-8404	Santa Cruz Biotechnology (Dallas, TX, USA)
p-NF-κB	sc-136548	Santa Cruz Biotechnology (Dallas, TX, USA)
IL-1β	sc-515598	Santa Cruz Biotechnology (Dallas, TX, USA)
BDNF	#47808	Cell Signaling Tech (Rosemont, IL, USA)
p-Akt	sc-514032	Santa Cruz Biotechnology (Dallas, TX, USA)
p-GSK-3β	sc-373800	Santa Cruz Biotechnology (Dallas, TX, USA)
p-Tau	sc-32275	Santa Cruz Biotechnology (Dallas, TX, USA)
p-CREB-1	sc-81486	Santa Cruz Biotechnology (Dallas, TX, USA)
BCl-2	sc-7382	Santa Cruz Biotechnology (Dallas, TX, USA)
BAX	sc-7480	Santa Cruz Biotechnology (Dallas, TX, USA)
Caspase-3	sc-56053	Santa Cruz Biotechnology (Dallas, TX, USA)
Goat-anti-rabbit IgG	#7074	Cell Signaling Tech (Rosemont, IL, USA)
Goat-anti-mouse IgG	#1724044	Bio-Rad (Richmond, CA, USA)

AChE, acetylcholinesterase; ChAT, choline acetyltransferase; SYP, synaptophysin; PSD-95, postsynaptic density protein 95; GAP-43, growth-associated protein 43; IDE, insulin degrading enzyme; ZO-1, zonula occludens-1; TLR4, Toll-like receptor 4; MyD88, myeloid differentiation primary response 88; p-JNK, phospho-c-Jun N-terminal kinase; p-IκB-α, phospho-inhibitor kappa B-alpha; p-NF-κB, phospho-nuclear factor kappa B; IL-1β, interleukin-1β; BDNF, brain-derived neurotrophic factor; p-Akt, phospho-protein kinase B; p-GSK-3β, phospho-glycogen synthase kinase-3 beta; p-Tau, phospho-tau; p-CREB-1, phospho-cyclic adenosine monophosphate (cAMP) response element binding protein; BCl-2, b-cell lymphoma 2 protein; BAX, BCl-2 associated X;
